# Authors’ Reply

**Published:** 2011

**Authors:** O E. Babalola, R E. Umeh, A O. Mahmoud

**Affiliations:** Rachel Eye Center, PO Box 4108, Garki Abuja, Nigeria; 1 Department of Ophthalmology, University of Nigeria, Enugu, Nigeria; 2 Department of ophthalmology, University of Ilorin Teaching Hospital, Ilorin, Nigeria

Sir,

Thank you for your enquiry regarding our publication and for allowing us to respond to the readers’ thoughtful comments. 1,2 The sensitivity (approaching 72%) and specificity (approaching 92%) of the test has been previously published.^3^ These values depend on a subjective cutoff point; the higher the cutoff, the higher the sensitivity and specificity of the test. However, we believe that there is adequate sensitivity and specificity for clinical practice.

Secondly, the initial cost for the program is not high. The software which usually comes on a floppy diskette can be easily loaded on a laptop. Hence there is minimal software and hardware cost.

Finally, the test was completed by 76% of the respondents, which is high when one considers that there were a number of visually impaired respondents in the study who could not clearly see the target, or were too old or infirm to comprehend or complete the test. This is remarkable as the test was carried on mainly in illiterate communities. In urban clinical practice, there are usually no problems with the completion of the test.
